# *CX3CR1* Is a Modifying Gene of Survival and Progression in Amyotrophic Lateral Sclerosis

**DOI:** 10.1371/journal.pone.0096528

**Published:** 2014-05-07

**Authors:** Alan Lopez-Lopez, Josep Gamez, Emilio Syriani, Miguel Morales, Maria Salvado, Manuel J. Rodríguez, Nicole Mahy, Jose M. Vidal-Taboada

**Affiliations:** 1 Biochemistry and Molecular Biology Unit, Department of Physiological Sciences I, Faculty of Medicine - IDIBAPS, University of Barcelona, Barcelona, Spain; 2 ALS Unit, Neurology Department, Hospital Universitari Vall d'Hebron - VHIR. Autonomous University of Barcelona, Barcelona, Spain; 3 Synaptic Structural Plasticity Lab, CIBIR, Logroño, Spain; 4 Centro de Investigación Biomédica en Red sobre Enfermedades Neurodegenerativas (CIBERNED, ISCIII), Barcelona, Spain; Inserm, France

## Abstract

The objective of this study was to investigate the association of functional variants of the human *CX3CR1* gene (Fractalkine receptor) with the risk of Amyotrophic Lateral Sclerosis (ALS), the survival and the progression rate of the disease symptoms in a Spanish ALS cohort. 187 ALS patients (142 sporadic [sALS] and 45 familial) and 378 controls were recruited. We investigated *CX3CR1* V249I (rs3732379) and T280M (rs3732378) genotypes and their haplotypes as predictors of survival, the progression rate of the symptoms (as measured by ALSFRS-R and FVC decline) and the risk of suffering ALS disease. The results indicated that sALS patients with *CX3CR1* 249^I/I^ or 249^V/I^ genotypes presented a shorter survival time (42.27±4.90) than patients with 249^V/V^ genotype (67.65±7.42; diff −25.49 months 95%CI [−42.79,−8.18]; p = 0.004; adj-p = 0.018). The survival time was shorter in sALS patients with spinal topography and *CX3CR1* 249^I^ alleles (diff = −29.78 months; 95%CI [−49.42,−10.14]; p = 0.003). The same effects were also observed in the spinal sALS patients with 249^I^–280^M^ haplotype (diff = −27.02 months; 95%CI [−49.57, −4.48]; p = 0.019). In the sALS group, the *CX3CR1* 249^I^ variant was associated with a faster progression of the disease symptoms (OR = 2.58; 95IC% [1.32, 5.07]; p = 0.006; adj-p = 0.027). There was no evidence for association of these two *CX3CR1* variants with ALS disease risk. The association evidenced herein is clinically relevant and indicates that *CX3CR1* could be a disease-modifying gene in sALS. The progression rate of the disease's symptoms and the survival time is affected in patients with one or two copies of the *CX3CR1* 249I allele. The *CX3CR1* is the most potent ALS survival genetic factor reported to date. These results reinforce the role of the immune system in ALS pathogenesis.

## Introduction

Amyotrophic lateral sclerosis (ALS), also known as Lou Gehrig's disease or *maladie de Charcot*, is the most common adult onset neurodegenerative motor neuron disease [1], [2]. Patients present progressive weakness, spasticity and amyotrophy due to a progressive degeneration of the motor neurons in the cortex, brainstem, and spinal cord. Involvement of the respiratory muscles, especially the diaphragm, leads to respiratory failure and death. Most patients consequently die within 5 years of diagnosis, although the spectrum of survival time is broad [1], [2].

There is as yet no known etiology for the disease in most patients. Epidemiological studies suggest that major genetic defects make fewer than 10% of ALS patients susceptible to the disease. Environmental factors have been suggested for populations with a higher than average incidence, e.g. professional soccer players, veterans of the 1991 Gulf War and the population of the Western Pacific. For the majority of the population, ALS is considered a multifactorial disease with multiple interactive pathogenic mechanisms [3]–[6]. This variability of pathogenic mechanisms may be the reason why ALS is a heterogeneous disorder from the clinical perspective, in terms of the age at onset, progression, initial topography and survival.

It is unknown why some patients with ALS deteriorate much faster or survive much less time than others. An important challenge to ALS research is to find out how endogenous factors modify the disease to account for these different disease courses. The discovery of new biomarkers associated with different rates of progression and survival could offer new insights into the pathophysiological determinants of disease progression in ALS [7], [8]

At least 15 GWAS have been published in ALS. The majority of these have made a major contribution to the discovery of new genes causing this disease [9]. Some risk loci have also been identified in some of these GWAS, although the role of many of them (FGGY, ITRP2 and DPP6) has not been replicated when they have been studied in other populations. In addition to these risk loci, some of these GWAS have sought genes influencing the phenotype. Examples include KIFAP3 and EPHA4. The age of onset has recently been reported as being modulated by a locus on 1p34.1 [10]. The involvement of neuroinflammation in the pathogenesis of ALS has been a subject of increasing interest in recent years. Neuroinflammation has been reported as a pathological hallmark of ALS [11]. Microglia activation and the crosstalk between immune cells appear to play a significant role in neuronal death in both *in vivo* and *in vitro* studies [12]–[14]. In ALS, microglial activation correlates with disease progression and symptoms, and might therefore modify the outcome of this devastating disease [15]–[19].

The *CX3CR1* gene (chemokine (C-X3-C motif) receptor 1, also known as Fractalkine receptor, OMIM: 601470) in the brain is only expressed by microglia [20] and it has been proposed as a key mediator of neuron-microglia interactions that is upregulated in many inflammatory conditions [21], [22]. CX3CR1 mediates microglia neurotrophic functions and its reduction would reflect an impaired microglial function [23]. Actually, *CX3CR1* signaling impairment has a direct influence on neurodegenerative diseases of the central nervous system (CNS) that course with neuroinflammation, microglia and/or t-cell recruitment [24]–[26]. Two functional variants (V249I and T280M) have been described in the *CX3CR1* gene [27], [28]. These variants affect the activity of the CX3CR1 protein and have been associated with several inflammatory diseases such as multiple sclerosis [29], Crohn's Disease [30], AIDS [31], age-related macular degeneration [32] and coronary artery disease [32]. However, there are controversial results and there is to date no evidence that CX3CR1 could be a relevant risk factor for these diseases using GWAS.

In this paper, we hypothesized that the functional variants V249I and T280M in the *CX3CR1* gene may modify the risk of suffering from ALS and the disease's outcome/prognosis. Here we report the association between these two *CX3CR1* variants and the survival time and the progression rate of the disease symptoms in a cohort of Spanish sporadic ALS (sALS) patients.

## Methods

### Study population and clinical data

A cohort of 223 ALS patients (wALS) meeting the World Federation of Neurology revised El Escorial criteria for laboratory-supported or definite ALS was included in this study [34]. A control cohort of 750 non-related and non-affected subjects was recruited from the Spanish National DNA Bank (Salamanca, Spain). The control and ALS patients were Spanish citizens of European origin.

The majority (n = 187) of ALS patients was clinically characterized, monitored and received quarterly follow-up in the Hospital Universitari Vall d'Hebron ALS Unit. Sex, age at onset, initial topography and survival time between the clinical onset of symptoms and either the indication of non-invasive ventilation, or tracheostomy ventilation, or the date of death were ascertained for each patient using the homogeneous criteria of a unique observer (Dr. Gamez) (see Table 1 for a detailed demographic description of the populations).

**Table 1:** Demographic data of the different groups analyzed.

		Controls	sALS	fALS	wALS (all)
**Subjects**	all^a^	378 (750)	142	45	187
**Sex**	Men	198 (52.4%)	75 (52.8%)	23 (51.1%)	98 (52.4)
	Women	180 (47.6%)	67 (47.2%)	22 (48.9%)	89 (47.6)
**Age**	all^c^	58.23±14.53	61.66±13.32	55.15±11.55	60.60±13.41
	range	28–99	27–91	36–80	28–91
	Men^c^	54.42±8.72	61.63±13.34	54.83±13.36	60.03±13.18
	Women^c^	62.44±18.11	61.70±13.39	56.03±9.76	60.25±13.18
**Age at onset**	All^c^	-	57.53±13.96	49.76±11.27	57.88±13.63
**Age at death**	all^c^	-	63.64±12.49	56.12±10.33	58.78±13.46
**Mortality**	all^b^	-	83 (58.5%)	25 (55.6%)	108 (57.8%)
**Topography**	Spinal^b^	-	102 (71.8%)	32 (71.1%)	134 (71.7%)
	Bulbar^b^	-	38 (26.8%)	13 (28.9%)	51 (27.3%)
	Respiratory^b^	-	2 (1.4%)	0 (0.0%)	2 (1.1%)

Statistics format: ^a^n (control cohort);

b n (%);

c mean ±SD.

The patients were classified as (i) familial or sporadic ALS, (ii) bulbar, spinal or respiratory onset, and according to (iii) the rate of progression and (iv) survival time. In order to classify the patients' rate of progression, the patients were decreasing ordered according the mean slope for ALSFRS-R decline and for FVC decline criteria. The distribution in tertiles was used to classify patients in three subgroups: rapid progression (subgroup P1, first tertil for ALSFRS-R and FVC values); normal progression (subgroup P2, second tertil for ALSFRS-R and FVC values); and slow progression (subgroup P3, third tertil for ALSFRS-R and FVC values).

Survival time was defined as the time between the onset of clinical symptoms and the date of death, or the date of tracheostomy ventilation, or the date when non-invasive ventilation of more than 22 h/day was required.

### DNA purification and Genotyping

Genomic DNA samples were extracted from whole blood samples of patients using QIAamp DNA Mini Kit (QIAGEN, USA) following the manufacturer's instructions.

The *CX3CR1* V249I (rs3732379) and T280M (rs3732378) alleles were genotyped using the KASPar SNP Genotyping system (KBioscience, UK) according the standard provider procedures.

The *CX3CR1* gene T280M and V249I variants were identified after amplification with V249I primers set (249_V: CTT CTG GAC ACC CTA CAA CG; 249_I:CCT CTT CTG GAC ACC CTA CAA CA; 249rev: GAG CTT AAG YGT CTC CAG GAA AAT CAT) and T280M primer set (280_T: GGC CCT CAG TGT GAC TGA GAC; 280_M: GGC CCT CAG TGT GAC TGA GAT; 280_rev: GAG AGG ATT CAG GCA ACA ATG GCT A). Fluorescence was measured at 25°C in a 7300 real time PCR System (Applied Bioscience, USA). Genotype calling was carried out using 7300 system SDS software v1.4 (Applied Bioscience, USA) and Klustercaller software (KBioscience, UK).

### Statistical Design, Analysis and Power

The study design consisted of a pragmatic, case-control, retrospective clinical study. The sample analyzed consisted of 223 ALS patients and 474 controls (two controls per case), matched for age and sex, who were randomly selected for each case to assess the risk of disease. *CX3CR1* V249I (rs3732379^CT^) and T280M (rs3732378^GA^) genotypes, or their haplotypes, were used as predictors of the progression rate of the symptoms, the survival time, the risk of suffering from ALS and the age at onset.

Associations for each SNP, the odds ratios and the 95% Confidence Interval (CI) were computed using generalized linear models (either for quantitative or binary traits), as implemented in SNPassoc package from R Software [35]. Analyses were done under 4 different inheritance models: dominant, recessive, additive and codominant. The best model was chosen using the Akaike information criteria (AIC) and the Hardy-Weinberg equilibrium (HWE) was calculated using Fisher's exact test implemented in the SNPassoc package. Ordinal logistic regression was carried out using SPSS software. Single marker analyses of disease risk were carried out using conditional logistic regression in R (package survival). Haplotype analyses of *CX3CR1* SNPs were performed with the *SNPassoc package*.

Single marker and haplotypes analyses were performed adjusted by sex and when necessary, stratified by topographic clinical onset. Bonferroni correction for 2 SNPs was used to correct for multiple comparisons. An additional factor of correction of 2.5 was applied to account for the use of 4 different genetic models [35], [36]. Using this criterion, the uncorrected level for statistical significance was set to p<0.011. The uncorrected and corrected p-values are shown.

Survival analysis was performed using Kaplan-Meier curves and COX regression (SPSS software) and multiple linear regression analysis (SNPassoc) including all the dead patients and those alive with either non-invasive ventilation for longer than 22 hours a day or ventilation by tracheostomy.

This study has a prior statistical power of 80% (alpha = 5%) for detecting a difference of 22 months in survival between groups of patients (main population survival = 49.27±41.6 months, minor allele frequency = 0.288), and a beta of 90% (alpha = 5%) for detecting a difference of 25,2 months. Concerning to the risk to suffer ALS this study have a power of 80% (alpha = 5%) for detecting an OR>1.7 between cases and controls.

### Ethics Statement

This genetic study was approved by the local IRBs at the Vall d'Hebron Research Institute and the Spanish National DNA Bank. The study has been conducted according to the principles set out in the Declaration of Helsinki. All patients gave their written informed consent to participation in the study in paper format, and a blood sample for genetic analysis was obtained from all of them. The categorization of the ALS patients was performed according to their clinical features (familial or sporadic ALS forms, bulbar or spinal or respiratory onset, rate of progression and survival time).

## Results

### *CX3CR1* variants and ALS susceptibility

A DNA sample for genetic analysis of a total of 187 ALS patients (wALS group) and 378 controls randomly selected from the 750 representatives of the Spanish population control cohort was obtained. 45 were familial ALS cases (fALS group), and 178 patients were sporadic cases (sALS group). We had full clinical details for all familial and sporadic cases. All the DNA samples were genotyped for the functional variants V249I (rs3732379) and T280M (rs3732378) of the *CX3CR1* gene. Both genetic variants were within the Hardy-Weinberg equilibrium in the different groups (wALS, sALS, fALS and controls, HWE (all groups) for V249I = 0.579; for T280M = 0.399; Table S1).

The *CX3CR1* variants 249I (rs3732379^T^ allele) and 280M (rs3732378^A^ allele) were assessed as genetic risk markers for ALS. Single marker and haplotype analysis showed no statistically significant differences between these variants in the controls and ALS cases of the wALS group in any of the 4 tested genetic inheritance models (Table S2). Genetic association analysis of the sALS or fALS groups also showed no statistically significant differences compared to the controls.

### *CX3CR1* variants and Survival

Single marker analyses associating variants in *CX3CR1* gene with the survival time of ALS patients, measured in months, were performed in the three ALS groups (wALS, sALS and fALS). The generalized linear models analysis showed that sALS patients with the *CX3CR1*-V249^I/I^ and V249^V/I^ genotypes had a shorter survival time (42.27±4.90) than patients with the 249^V/V^ genotype (67.65±7.42) under a dominant and additive inheritance model (diff = −25.49; 95%CI [−42.79, −8.18]; p = 0.004; corrected p-value = 0.018; Table 2). The dominant model had the lower AIC. In the wALS and fALS groups, this difference was not significant (Table 2).

**Table 2:** Single marker analysis for survival (in months) in different types of ALS patients assuming a dominant model.

Genetic variant	Group	Genotype	n	Survival (Median ±SEM)	Difference (95%CI)	p-value	Corrected p-value
**V249I**	**wALS**	V/V	74	65.57±6.35	0.0		
		V/I+I/I	70	50.44±6.141	−14.48 (−32.33, 3.36)	0.112	0.504
	**sALS**	V/V	51	67.65±7.42	0.0		
		V/I+I/I	56	42.27±4.90	−25.49 (−42.79, −8.18)	**0.004**	**0.018**
	**fALS**	V/V	23	60.96±12.31	0.0		
		V/I+I/I	14	83.14±24.12	27.00 (−21.28, 75.28)	0.273	1.000
**T280M**	**wALS**	T/T	108	58.17±4.74			
		M/T+M/M	36	58.41±11.45	−0.19 (−20.46, 20.84)	0.985	1.000
	**sALS**	T/T	78	57.56±5.37	0.0		
		M/T+M/M	29	45.76±8.27	−11.82 (−31.80, 8.16)	0.246	1.000
	**fALS**	T/T	30	59.73±9.96	0.0		
		M/T+M/M	7	110.57±45.22	51.84 (−5.65, 109.3)	0.077	0.347

Genotype correspondence: V = rs3732379^C^ allele, I = rs3732379^T^ allele, T = rs3732378^G^ allele, M = rs3732378^A^ allele.

**Figure 1:**
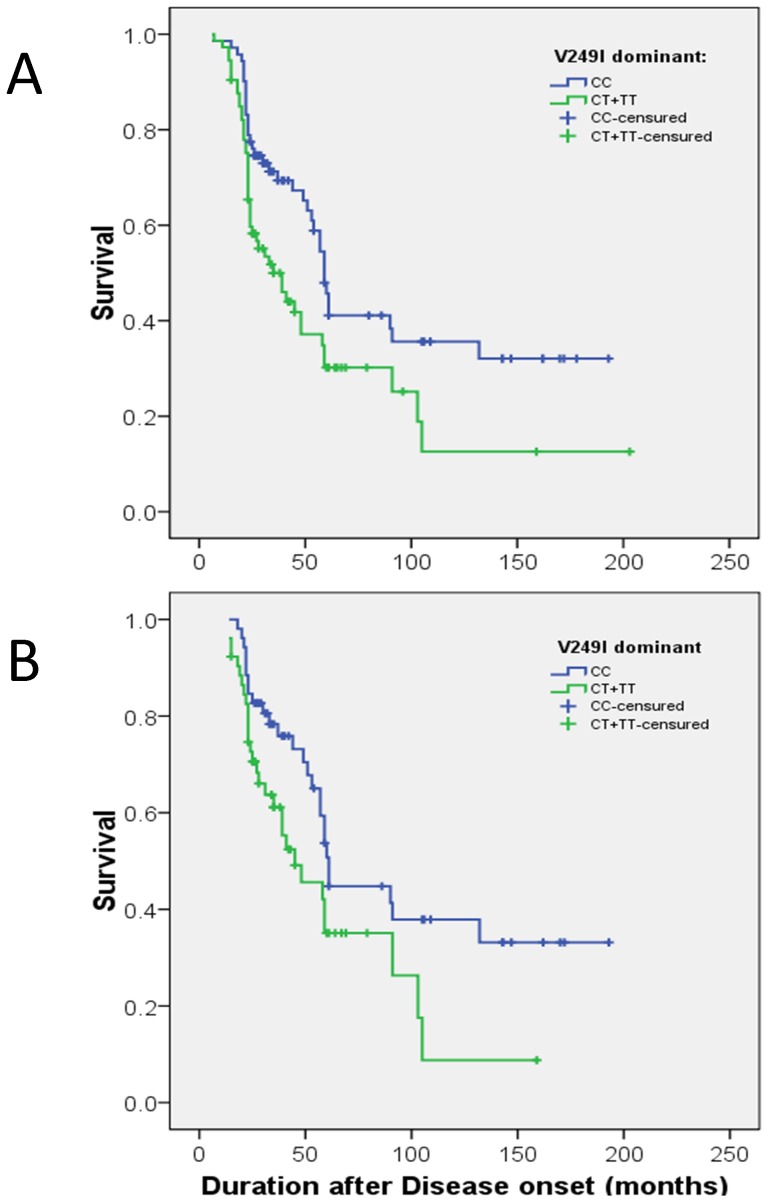
Kaplan–Meier survival curves for CX3CR1-V249I genotypes according to a dominant genetic model in the sALS group (A) and the sALS group with only spinal topography (B). The green line is for V/I+I/I genotypes, and the blue line is for the VV genotype.

We grouped sALS patients under a dominant model and performed a Kaplan Meyer Survival curve analysis. The results evidenced statistical differences between *CX3CR1* 249^V/V^ (median = 59.0 months, 95%CI [53.7, 64.3]) and 249^V/I^ or 249^I/I^ (median = 35.00 months, 95%CI [21.1, 48.9]) patients (long Rank = 6.357; HR = 1.72 95%CI [1.15, 2.67]; p-value = 0.014; Figure 1A).

We subsequently carried out a genetic analysis stratifying for the topography of the disease onset (classic spinal, bulbar and respiratory). Spinal sALS patients with *CX3CR1* 249^V/I^ and 249^I/I^ genotypes had a shorter survival time than patients with 249^V/V^ genotype (diff −29.78, 95%CI [−49.42, −10.14], p-value = 0.003). This effect was not statistically significant in the spinal wALS group. For sALS patients with spinal topography, the survival curves also showed differences between 249^V/V^ patients (median = 61.00 months, 95%CI [56.7, 65.3]) and 249^V/I^ and 249^I/I^ (median = 45.0 months, 95%CI [22.9, 67.1]) patients under a dominant model (long rank = 4.507; HR = 1.76 95%CI [1.03, 3.01]; p-value = 0.038; Figure 1B).

The haplotype analysis showed that sALS patients with the 249^I^–280^T^ haplotype presented a shorter survival time than patients with the 249^I^–280^M^ or 249^V^–280^T^ haplotypes (Table 3) under an additive genetic inheritance model. The spinal sALS patients with the *CX3CR1* 249^I^–280^M^ haplotype (diff −27.02, 95%CI [−49.57, −4.48], p-value = 0.019, model p-Value = 0.0001) and patients with the *CX3CR1* 249^I^–280^T^ haplotype (diff −22.9295%CI [−46.13, 0.29] p-value = 0.053; model p-value = 0.0001) also presented a shorter survival time.

**Table 3:** Haplotype analysis for survival in different types of ALS patients.

Group	Haplotype V249I-T280M	Freq.	Survival (Median ±SEM)	Difference (CI 95%)	p-value	Corrected p-value	model p-value
**wALS**	**V-T**	0.735	65.39±6.01	0.0 (r_h)			**0.0001**
	**I-T**	0.136	44.19±9.82	−21.20 (−40.45, −1.96)	**0.031**	0.140	
	**I-M**	0.129	59.11±10.02	−6.05 (−25.69, 13.59)	0.546	1.000	
**sALS**	**V-T**	0.722	71.50±15.19	0.0 (r_h)			**0.0001**
	**I-T**	0.139	41.71±10.22	−26.79 (−46.82, −6.76)	**0.009**	**0.041**	
	**I-M**	0.139	53.44±9.61	−18.06 (−36.90, −0.78)	0.062	0.279	
**fALS**	**V-T**	0.769	26.36±37.30	0.0 (r_h)			**0.0001**
	**I-T**	0.128	19.45±23.4	−6.91 (−52.78, 38.95)	0.768	1.000	
	**I-M**	0.103	67.38±28.3	41.02 (−14.39, 96.42)	0.147	0.662	

r_h = reference haplotype (most prevalent). V = rs3732379^C^ allele, I = rs3732379^T^ allele, T = rs3732378^G^ allele, M = rs3732378^A^ allele.

### *CX3CR1* variants and disease progression

Single marker analyses for the rate of progression of the ALS symptoms were carried out in the wALS, sALS and fALS groups. An increased frequency of patients with *CX3CR1* 249^I/I^ and 249^V/I^ genotypes was found in the wALS subgroup with a rapid progression rate, under a dominant inheritance model (OR = 1.89; subgroup P1 vs P3) (Table 4). This effect was higher (OR = 2.58) in the sALS subgroup with a rapid progression rate. Patients with the *CX3CR1* 249^V/V^ genotypes were most common in the slow progression rate subgroups (P3 vs P1 in wALS and sALS) (see Table 4). In the fALS group, this difference in the progression rate of the disease's symptoms did not reached statistical significance.

**Table 4:** Single marker analysis for progression rate of the disease symptoms in different types of ALS patients (Dominant model).

			Progression subgroup (n)			Effect of P1compared to P3 subgroup			
Genetic variant	Group	Genotype	P1	P2	P3	OR (95%CI)	p-value	Corrected p-value	Model p-value
**V249I**	**wALS**	V/V	27	41	29	0.0			
		V/I+I/I	38	36	17	1.89 (1.07, 3.37)	**0.029**	0.131	**0.028**
	**sALS**	V/V	19	32	20	0.0			
		V/I+I/I	34	28	11	2.58 (1.32, 5.07)	**0.006**	**0.027**	**0.005**
	**fALS**	V/V	8	9	9	0.0			
		V/I+I/I	4	8	6	0.65 (0.20, 2.23)	0.476	1.000	0.363
**T280M**	**wALS**	T/T	47	55	37	0.0			
		M/T+M/M	18	21	9	1.48 (0.77, 2.84)	0.234	1.000	0.473
	**sALS**	T/T	36	43	26	0.0			
		M/T+M/M	17	17	5	1.90 (0.90, 4.03)	0.093	0.419	0.083
	**fALS**	T/T	11	12	11	0.0			
		M/T+M/M	1	4	4	0.60 (0.15, 2.45)	0.474	1.000	0.741

Progression rate groups: Fast (P1), Normal (P2), slow (P3). Genotype correspondence: V = rs3732379^C^ allele, I = rs3732379^T^ allele, T = rs3732378^G^ allele, M = rs3732378^A^ allele.

In the subgroup of spinal sALS patients with fast progression, the 249^I/I^ and 249^V/I^ genotypes were more frequent than the 249^V/V^ genotype (I/I+I/V = 18 vs V/V = 9) under a dominant inheritance model. Conversely, in the subgroup of spinal sALS with slow progression (P3), the 249^V/V^ genotype was more common than the 249^I/I^ and 249^V/I^ genotypes (I/I+I/V = 9, V/V = 19). The OR for the *CX3CR1* V249I variant for the fast progression of the disease symptoms was 2.85 (95%CI [1.27, 6.38], p = 0.011).

### *CX3CR1* variants and age at onset and site of clinical onset

The results of the genetic analyses of the *CX3CR1* variants using a single marker or haplotype did not show any statistically significant difference for an earlier age at onset of ALS, or predict the site of clinical onset in any of the analyzed groups (wALS, sALS and fALS).

## Discussion

In this study, we report on the association of a shorter survival time and faster progression rate of the disease's symptoms with the *CX3CR1* V249I genetic variant (rs3732379^T^ allele) among the patients of our ALS series in the sporadic ALS group. The sALS patients with the *CX3CR1* 249^I/I^ or 249^V/I^ genotypes have a shorter survival time than patients with 249^V/V^ genotype. The survival time was shorter in the group of sALS patients with spinal topography and 249^I^ alleles, and with the 249^I^–280^M^ haplotype. A higher frequency of the *CX3CR1* 249^I^ variant was also found in patients with a fast progression of symptoms. Taken together, these results suggest that the *CX3CR1* acts as a disease-modifying gene in sALS patients, and point to its role in ALS pathogenesis.

The *CX3CR1* is the most potent ALS survival genetic factor reported to date. Using GWAS analysis, The KIFAP3 [37] EPHA4 [38], the UNC13A [39] and the SLC11A2 genes [40] have recently been described as factors modifying survival in USA and European populations. The UNC13A gene, associated with a reduction in survival of 5 to 10 months, encodes for a protein that regulates the release of neurotransmitters at neuromuscular synapses [39]. The SNP rs1541160 in the *KIFAP3* gene, a gene related with the immune system pathway, reduces the survival of ALS patients in 14–14.9 months [37]. The SLC11A2 gene, that encodes the divalent metal transport 1 (DMT1) mediating iron transport in cerebral endosomal compartments, has been associated with a shorter duration of ALS disease of 17 months (HR 1.5)[40]. In our study, the *CX3CR1* 249^I^ allele (under a dominant genetic model) reduces survival time by a median of 25.49 months (95%CI [−42.79, −8.18] in the sALS group, which is much higher than those observed for the *UNC13A* rs12608932 and *KIFAP3* rs1541160 polymorphisms. A comparison of the hazard ratios (HR) shows that the risk of survival reduction is higher for patients with *CX3CR1* 249^I^ than for *UNC13A* rs12608931^A/A, A/C^ variants (1.72 vs 1.28). Other genes, such as *SMN1* and *SMN2*, have also been considered as possible survival risk factors, with differing results [41]–[43].

This is the first report that investigates the influence of genetic factors on the progression rate of the disease symptoms. Our results identified *CX3CR1* 249^I^ allele as a factor that modifies ALS clinical progression. When analyzing the effect of this genetic variant on the FVC and ALSFRS-R slopes, the key non-biological markers of the disease's progression, the OR for the risk of fast progression was 2.3 for *CX3CR1* 249^I^ carriers. When stratifying for the site of clinical onset, sALS patients with spinal onset with the *CX3CR1* 249^I^ alleles showed an increased risk of fast progression of 2.6 compared to homozygous 249^V^ patients. This is clinically relevant for identifying patients at high risk of requiring vital support measures, such as mechanical ventilation or gastrostomy tube feeding.

In our cohort, we found three sALS cases and five fALS cases with C9orf72 expansions distributed in the fast, the normal and the low disease progression subgroups, and the short and normal survival subgroups. In our sALS cohort, the expansions in the C9orf72 gene did not influence the results of CX3CR1 as regards survival and disease progression.

The effect of the *CX3CR1* variants on survival time and progression rate was observed in the sALS group and not in the fALS group. The genetic heterogeneity observed in fALS is the major genetic factor influencing the resulting phenotype. For example, the *p.D91A* (D90A in the old nomenclature) variant in the SOD1 gene in a homozygous state has been reported as predicting a long survival time (>30 years) in ALS1 Scandinavian patients [44], and a similar effect has been observed in the *p.G38R* (G37R in the old nomenclature) variant worldwide [45]. By contrast, the *p.A5V* (A4V in the old nomenclature) variant has been associated with survival times shorter than 12 months [45]. Mutations in ALSIN gene also predicted juvenile onset [46]. Our fALS series included four symptomatic family members carrying p.G38R with a very slow progression and long survival (144 months) [47]. Consideration of the phenotype heterogeneity of these and other mutations in the 14 major genes causing fALS may explain why the *CX3CR1* variants are not associated with survival in our fALS series. Actually, this is one of the reasons why fALS group is rarely included in ALS progression biomarker studies, as its heterogeneity could affect the statistical association of sALS.

In the CNS the *CX3CR1* gene is only expressed by microglia, being a gene marker for microglia [20]. The V249I and T280M variants in the human *CX3CR1* affect the functionality and activity of the *CX3CR1* protein [27], [28], [33]. The 249^I^ variant has been associated with reduced number of fractalkine binding sites and reduced fractalkine binding affinity on peripheral blood mononuclear cells, resulting in a loss of function [33]. The mechanisms underlying 280^M^ effects are not clear. Some studies have reported reduced cell-to-cell adhesion under physiological conditions of the 280^M^ allele [28], [31], [33], while other authors have described 280M as promoting excess adhesion [27]. Despite these controversial results, this variant has been associated with an atheroprotective effect [48]. Our results indicate that the contributions of allele 249I and 280^M^ in the CX3CR1 haplotypes are associated with decreased survival in sALS with spinal topography. However the presence of the single allele 249^I^ is responsible for the association with survival in the sALS group. Our results indicated neither an additive effect of 249^I^ and 280^M^ alleles, nor a protective effect like that reported for cardiovascular disease [28], [49].

In animal models, *Cx3cr1* ^−/−^ knockout mice inbred with the SOD1^G93A^ transgenic ALS model present a worsened disease outcome, more extensive neuronal loss and increased microglial activation [22]. *CX3CR1* performs neurotrophic functions and its reduction would reflect an impaired microglial function [50]. Thus, decreased CX3CR1 activity may contribute to ALS pathogenesis in part by enhancing inflammatory activity of microglia, which rather than initiate motor neuron degeneration would accelerate the disease progression.

This study is well powered (beta = 90%, alpha = 5%) for detecting changes in the survival in sALS patients with different CX3CR1 genotypes. However, these findings should be confirmed in further studies with larger cohorts of ALS Spanish patients and in other populations. On the other hand, the sample size is a major limitation to evaluate the risk to suffer ALS. This study has enough power (beta = 80% alpha = 5%) to detect a risk effect if OR>1.7 (under a dominant genetic model) or if OR>2.2 (under a recessive model). Our risk analysis for CX3CR1 variants indicates that the OR is about 0.88 with 95%CI [0.64 −1.22]. If CX3CR1 could be associated with the risk of suffer ALS, this means post-hoc power of only 11.6%. Based on our results and on public results of GWAS for ALS, these findings could indicate that the CX3CR1 rs3732378 and rs3732379 are probably not a risk factor for suffering ALS. Alternatively, these two SNPs in CX3CR1 could be a low risk factor for ALS (OR<1.22).

## Conclusions

Our results indicate that *CX3CR1* is a modifying gene in sporadic ALS, which affects the progression rate of the symptoms and the survival time in patients with one or two copies of the *CX3CR1* 249^I^ allele. *CX3CR1* V249I and T280M variants may therefore be used as genetic markers in the clinical setting as prognostic factors for ALS survival and the progression of the disease. Our results are clinically relevant and reinforce the role of the immune system in ALS pathogenesis.

## Supporting Information

Table S1 **p-values for the HWE test in the different groups.**
(DOC)

Table S2 **Single marker analysis p-values for risk of suffering ALS by different genetic models.**
(DOC)
