# Supplementary material for: CX3CR1 Is a Modifying Gene of Survival and Progression in Amyotrophic Lateral Sclerosis
Source: PLoS One. 2014 May 7;9(5):e96528. doi: 10.1371/journal.pone.0096528 (PMC4013026; doi:10.1371/journal.pone.0096528)
Supplement: Table S1 — p-values for the HWE test in the different groups. (DOC) [file pone.0096528.s001.doc]

**Table S1**. p-values for the HWE test in the different groups

| **Group** | **CX3CR1 variant** | **Control** | **Cases** |
| --- | --- | --- | --- |
| **wALS** | V249I | 0.257 | 0.088 |
|  | T280M | 0.844 | 0.209 |
| **sALS** | V249I | 0.257 | 0.059 |
|  | T280M | 0.844 | 0.309 |
| **fALS** | V249I | 0.257 | 0.683 |
|  | T280M | 0.844 | 1.000 |

T280M= rs3732378, V249I= rs3732379
