# Supplementary material for: CX3CR1 Is a Modifying Gene of Survival and Progression in Amyotrophic Lateral Sclerosis
Source: PLoS One. 2014 May 7;9(5):e96528. doi: 10.1371/journal.pone.0096528 (PMC4013026; doi:10.1371/journal.pone.0096528)
Supplement: Table S2 — Single marker analysis p-values for risk of suffering ALS by different genetic models. (DOC) [file pone.0096528.s002.doc]

**Table S2.** **Single marker analysis p-values for risk of suffering ALS by different genetic models**

|  |  |  | **Genetic model** | | | |
| --- | --- | --- | --- | --- | --- | --- |
| **Group** | **CX3CR1 variant** | **case-control (n)** | **Dominant** | **Recessive** | **Additive** | **Codominant** |
| **wALS** | V249I | 186-372 | 0.654 | 0.198 | 0.387 | 0.432 |
|  | T280M | 185-370 | 0.468 | 0.122 | 0.302 | 0.275 |
| **sALS** | V249I | 142-284 | 0.948 | 0.119 | 0.596 | 0.267 |
|  | T280M | 142-284 | 0.753 | 0.225 | 0.560 | 0.478 |
| **fALS** | V249I | 44-88 | 0.232 | 0.970 | 0.323 | 0.465 |
|  | T280M | 41-82 | 0.261 | 0.186 | 0.192 | 0.274 |

T280M= rs3732378, V249I= rs373237
